# Hexane extract of *Persea schiedeana* Ness as green corrosion inhibitor for the brass immersed in 0.5 M HCl

**DOI:** 10.1038/s41598-024-56793-w

**Published:** 2024-03-18

**Authors:** Genoveva BustosRivera-Bahena, A. M. Ramírez-Arteaga, Hugo A. Saldarriaga-Noreña, A. K. Larios-Gálvez, José G. González-Rodríguez, M. Romero-Aguilar, Roy López Sesenes

**Affiliations:** 1 CIICAp-IICBA, Universidad Autónoma del Estado de Morelos, Av. Universidad 1001, Chamilpa, 62209 Cuernavaca, Mexico; 2 FCQeI, Universidad Autónoma del Estado de Morelos, Av. Universidad 1001, Chamilpa, 62209 Cuernavaca, Morelos, Mexico; 3 Centro de Investigaciones Químicas-IICBA, Universidad Autónoma del Estado de Morelos, Av. Universidad 1001, Chamilpa, 62209 Cuernavaca, Mexico

**Keywords:** Corrosion inhibitor, Brass, Electrochemical analyses, *Persea schiedeana* Ness, Green corrosion inhibitor, Electrochemistry, Corrosion

## Abstract

The hexane extract of *Persea schiedeana* Ness (PSN) was analyzed as corrosion inhibitor for the brass surface immersed in 0.5 M HCl. Fourier-transform infrared spectroscopy and a gas chromatographic (GC) and mass spectrometric (MS) were used to identify the PSN extract’s functional groups and compound constituents. The functional groups identified were ${\text{C}}{\text{H}}_{3}$ and ${\text{C}}{\text{H}}_{2}$ functional alkyl groups, $\text{C=O}$ stretching vibration of aldehydes, ketones, and carbonyl groups. The GC/MS determined the presence of fatty acids in the PSN extract, where palmitic acid, oleic acid, and ethyl oleate were the major constituents. Electrochemical characterizations were conducted to observe the effect of PSN as corrosion inhibitor on the brass surface. The *R*_p_ and *R*_n_ calculated from EIS and ENA give the same behavior. Based on the OCP behavior, it was determined that the PSN works as a mix inhibitor, affecting both anodic and cathodic reactions. The corrosion current density (*I*_corr_) suggests that the extract of PSN reduces the corrosion rate of the brass with efficiencies above 90% for all concentrations. The efficiency obtained for each PSN concentration was attributed to forming a corrosion scale of ${\text{CuO}}$ and ${\text{C}}{\text{u}}_{2}{\text{O}}$, which reacted with the carboxyl group to form copper carboxylates on the metal surface.

## Introduction

Natural product extracts have been proposed in the last years as an alternative to be used as corrosion inhibitors due to having their chemical compounds such as aromatic rings, ${\text{O}}{\text{H}}^{-}$ groups, and heteroatoms (N, O, S, and P)^1,2^ which make them an excellent alternative to replace the synthetic organic and inorganic compounds currently used as corrosion inhibitor since well know that they can reduce the corrosion rate due to in their chemical structure exist the compounds mentioned before^3^. Also, the extraction methods used to obtain the active compounds are cheap and easy to carry out, such as Soxhlet, maceration, hydro distillation, and so on^4–6^.

Several metal surfaces are exposed to corrosive environments. Some modifications in their microstructure have been developed to reduce the aggressive effect on their surface by adding intermetallic elements, heat treatments, coating applications, organic and inorganic inhibitors, and so on. They have been evaluated using natural products as corrosion inhibitors, e.g., stainless steel, aluminum, aluminum alloys, copper, and copper alloys, which had reported low corrosion rates in different corrosion media (alkaline, acid, and neutral)^7^. One mechanism action reported that explains the low corrosion rate observed in the presence of natural products is their adsorption on the metal surface, which could be affected by the inhibitor concentration since a concentration above or below the optimal can lead to desorb it^8^. Other factors that could affect the inhibition efficiency of the natural products are the chemical compound extracted, the type of metal, temperature, the extraction method, and even the solvent used to extract the active compounds.

Although the studies using natural products are extensive, some fruits have yet to be explored, such as *Persea schiedeana* Nees, a fruit tree endemic to Mesoamerica. In México, it is typically known as chinin or pagua in the Veracruz and Chiapas regions. It belongs to the Lauraceae family, such as avocado (*Persea americana* Mill), which had been analyzed as a corrosion inhibitor by other authors^9–11^. *Persea schiedeana* Nees’s case has not been analyzed still as a corrosion inhibitor alternative. Even so, a few analyses were done by some authors, reporting that its chemical compositions found the presence of Fatty acids such as Oleic acid and Palmitic acid, as phenolic and antioxidant activities had been reported^12–14^. Then, most studies have been conducted to evaluate the nutritional value of the PSN or its agro-industrial potential to increase its production^15^. However, in recent dates, just one study has proposed its use as biodiesel, where López-Yerena et al*.* proposed that PSN can be a potential source to be transformed into biodiesel by alkaline transesterification fund moreover free fatty acids (8.36 ± 1.35%) and the main constituent of the biodiesel were methyl oleate (53.12%) and methyl palmitate (25.74%)^16^. Based on the latter, the present research could apport a new source to get a suitable corrosion inhibitor using wastes from the Agricola activity, explained based on constituents found in the IR and GC/MS analyses, how the *Persea schiedeana* Nees could act as a possible inhibitor on metal surface corrosion.

The content reported for the *Persea schiedeana* Nees includes ${\text{O}}{\text{H}}^{-}$ groups and aromatic rings, suggesting that PSN could be an excellent alternative to inhibit the negative effect of some corrosive environments on metal surfaces such as brass, which generally has been tested using organic and inorganic synthetic compounds which act forming complexes in active sites of the brass surface^17^. With the latter, the present research was focused on analyzing the inhibition activity of the hexane extract of *Persea schiedeana Nees* on the brass surface immersed in 0.5 M HCl, carrying out electrochemical analyses to estimate the corrosion rate. Also, a Fourier transform infrared (FT-IR) spectroscopy was done to identify the functional groups in the extract, such as alcohol groups, carbonyls, alkanes, and aromatics, which could be adsorbed on the surface brass, preventing the corrosion increases. Gas chromatography/mass spectrometry (GC/MS) was used to identify primary and secondary components in the hexane extract of *Persea schiedeana* Ness.

## Experimental procedure

### Materials

#### Preparation of the extract as corrosion inhibitor

To prepare the *Persea schiedeana* Nees extract, the natural row material was collected from a local market in Papantla, Veracruz México, during July and August. The part of the fruit used to prepare the extract was the seeds, which were cleaned with water. The hexane was used as a solvent for extracting the Persea schiedeana Nees since it is used in the industry for extracting oil, flavoring, and functional compounds. Moreover, it increases the extraction efficacy. Also, it is used with Soxhlet extraction as a green extraction method. The excess water was dried with a wipe and left in the shade for 1 week to dry completely. Once the seeds were dried completely, they were ground up using a blender first, and a ceramic mortar was used to end the ground-up process to obtain a finer size of the seeds. The powdered seed of Persea schiedeana Nees was previously weighted for use in a Soxhlet extractor with a capacity of 500 ml; the quantity of powdered seed employed in each extraction cycle was 50 mg, and it was placed into a thimble in the extraction chamber. After that, the hexane was poured into a distillation flask, and the reflux condenser was coupled to the chamber extract. The hexane in the distillation flask was heated to evaporate at 60 °C in a heating mantle. A refrigerant circulator system was used to ensure the condensation of the hexane and its drip down in the thimble; the extraction process was repeated several times (5 × 300 ml). The extract obtained (200 mg) was diluted in 10 ml of ethanol gauged with distiller water at 100 ml to prepare the inhibitor. After that, aliquots were prepared based on the test concentrations (100, 200, 300, 400, and 500 ppm) diluted in a solution of 0.5 M HCl as the corrosive medium.

#### Legislation of experimental research

The collection of plant material complies with relevant institutional, national, and international guidelines and legislation. All authors confirm that all methods followed the relevant guidelines in “Methods” section.

#### Metal surface preparation

A brass rod was used as a specimen for the test (Table 1), and from it, several samples were cut with an exposed area of 1 cm^2^ at 1 cm length. The samples were ground using emery paper grade 150, 300, 500, 600, 800, and 1000 to obtain a flatter surface free of oxide products based on ASTM-G1-03. After this, the samples were rinsed with acetone and dried with hot air. A copper wire was welded to each sample to make the measurements and ensure an electric path between the samples and the equipment. Then, the samples were encapsulated with epoxy resin to warrant an exposed area of 1 cm^2^. To remove the excess resin from the exposed surface, they were again ground and cleaned following the procedure previously described.

**Table 1:** Chemical composition of the Brass.

Element	Cu	Pb	Al	Fe	Ni	Sn	Mn	Zn
wt%	63	2.8	0.05	0.1	0.3	0.1	0.1	Balance

### Electrochemical tests

To determine the effect of the hexane extract of *Persea schiedeana* Nees in mitigating the corrosion rate on the brass surface, electrochemical tests such as electrochemical impedance spectroscopy (EIS), potentiodynamic polarization (PP) curves, and electrochemical noise (EN) were carried out. Before that, the open circuit potential (OCP) was measured.

The OCP was measured by 3700 s to observe the *E*_corr_ behavior without and with the inhibitor addition. The EIS was done following the procedure described on ASTM G 106, applying a sinusoidal amplitude of 10 mV in a frequency range from 10^4^ to 10^–2^ Hz concerning OCP. The PP curves were done by applying a start potential scan from − 1000 to 1000 mV with a sweep rate of 60 mV min^−1^ vs. OCP. The EN was conducted according to the ASTM G 102, recording 1023 points each second for both current and potential.

The electrochemical cell to carried out electrochemical analyses were conducted in a conventional cell of three electrodes using as working electrode (WE) the brass samples embedded in epoxy resin, a silver/chloride-silver electrode (Ag/AgCl) as reference electrode (RE), and a platinum wire as auxiliary electrode (AU). In the case of the EN, two identical brass samples were used as working electrode pairs (WE_1_ and WE_2_), and a platinum wire was used as RE.

All measurements were done at least three times to ensure reproducibility in a Gamry Interface 1010E Potentiostat, previously calibrated using a 2 kΩ calibration cell. Zview software version 3.0 was used to find the electrical circuit more appropriate based on the corrosion phenomena observed in the EIS results. To fit the electrical circuit model with the EIS experimental data, each element proposed was simulated using a freedom setting, fitting only positive values. The equivalent circuit proposed was submitted at different assays to reach a Chi-square less than 10^–3^, which indicates good fitting accuracy. To obtain approach values, an instant fit element was used to analyze by frequency range, starting from high to middle frequency and after simulating from middle to low frequencies. At the beginning of the simulation with the equivalent circuit, some data such as R_ct_, R_ox_, and n were fixed to estimate the adequate CPE.

### Chemical characterization

A Fourier-transform infrared (FT-IR) spectroscopy was used to identify the functional groups contained in the hexane extract of Persea schiedeana Nees (PSN) with a Thermo Scientific Nicolet 6700 in a region of 500–4000 cm^−1^. 200 µl of the extract mixed in 1.5 ml of distilled water was used to carry out the analyses and placed in a sample holder for liquids at a resolution of 2 cm^−1^. Also, a gas chromatographic (GC) 6890N (Agilent Technologies, San Francisco, CA, USA) and mass spectrometric (MS) 5975 (Agilent Technologies) were used to analyze the extract. The procedure used to carry out the GC/MS was as follows: the samples were injected (2 µl) automatically at 250 °C and separated through an hp5-ms: 5%phenyl-95%methylpolisiloxane (30 m × 0.25 µm). The carrier gas was helium (purity 99.99%), and the flow rate was maintained constant at 1 ml min^−1^. The oven temperature started at 40 °C and was gradually increased to 250 °C for 5 min, at a rate of 10 °C/min^−1^ after it was raised to 285 °C for 20 min, at a rate of 10 °C min^−1^. The mass spectrums were obtained by electronic impact (70 EV) and quadrupole mass analyzer. The scan mode (40–500 uma) was used to obtain the m/z, which was used to identify each compound.

### Scanning electron microscopy (SEM) and atomic force microscopy (AFM)

A scanning electron microscope LEO 1550VP with an emission gun and detector for energy-dispersive X-ray analysis was used to observe the corrosion product morphologies without and with PSN after being immersed in 0.5 M HCl. To improve the conductivity on the corroded surface, the samples were previously evaporated with gold (Au). To determine the roughness of the surface on the brass surface after being exposed to the corrosive media, an atomic force microscope from Park Systems XE-Bio, provided with an HQ: NSC16/No Al cantilever, was used in intermittent contact mode (tapping mode) with a selected area of 256 × 256 pixels with a scan rate of 0.3–0.5 Hz, at a gain between 3 and 5 with an oscillation amplitude between 4 and 5 nm. The images obtained were analyzed using Gwyddion 2.58 software.

## Results and discussions

### Identification of chemical compounds in PSN by FT-IR and GC/MS

**Figure 1:**
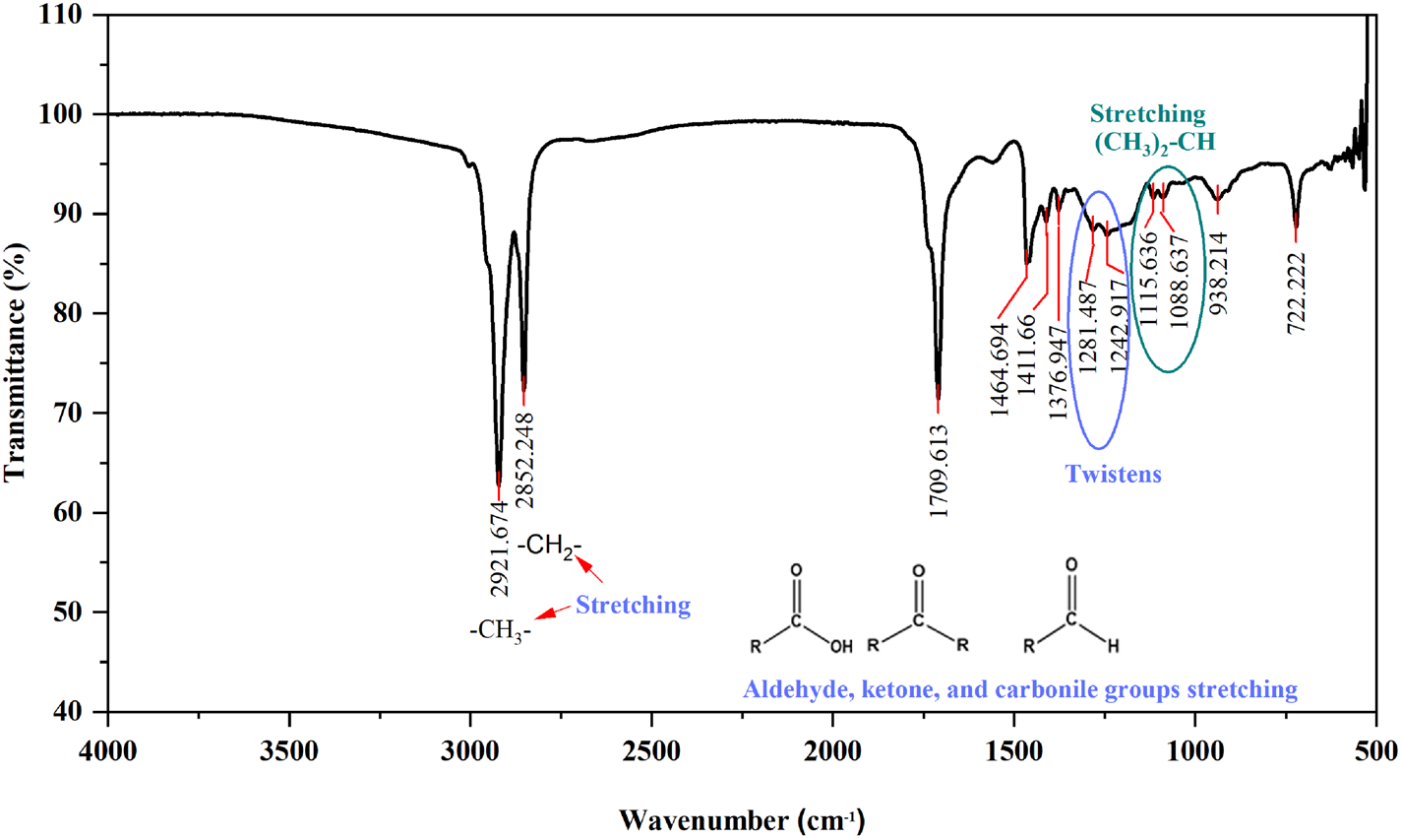
FT-IR for the hexane extract of Persea schiedeana Nees (PSN).

The FT-IR carried out on the *Persea schiedeana* Nees (PSN) is shown in Fig. 1. The highest intensity absorbance peaks for the PSN extract are localized at 2921, 2852, 1709, 1464, 1411, 1376, 1281, 1242, 1115, 1088, 938 and 722 cm^−1^. The absorbance peak between 2921 and 2852 cm^−1^ corresponds with the C–H stretching vibration of the ${\text{C}}{\text{H}}_{3}$ and ${\text{C}}{\text{H}}_{2}$ functional alkyl groups^18^. The strong peak at 1709.613 cm^−1^ is assigned to the $\text{C=O}$ stretching vibration of aldehydes, ketones, and carbonyl groups found in flavonoids and fatty acids^11^. The weak peaks between 1464 and 1376 cm^−1^ correspond with the C–H bending vibration of the aldehyde group^6^. Meanwhile, the weak peaks between 1115 and 1088 cm^−1^ correspond with the C–O stretching vibration of the ether and ester group. The small peak at 722 cm^−1^ corresponds to the ${\text{CH}}$ out-of-plane bending vibrations in the PSN constituents^19,20^.

### Chemical constituents of PSN via GC/MS

**Figure 2:**
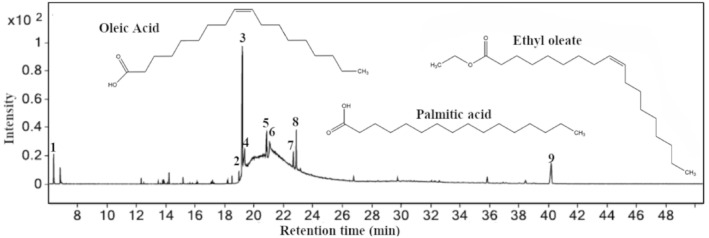
GC/MS chromatogram of hexane extract of PSN.

Figure 2 shows the GC/MS chromatogram of the hexane extract of PSN constituents, which was carried out by comparing their spectral fragments with available database libraries Wiley and NIST. Table 2 lists essential oils and fatty acid constituents identified in the hexane extract based on the retention time (RT). The primary constituents of them correspond with Oleic Acid (25.13%), Humulane-1,6-dien-3-ol (25.39%), Undecanoic acid, ethyl ester (27.85%), n-Hexadecanoic acid (62.25%), and Ethyl Oleate (64.32%). The last two compounds represent the main constituent in the PSN extract in agreement with findings reported by López-Yerena et al*.*, who reported fatty acids in the analysis done by NMR spectroscopy where the Hexadecanoic and octadecenoic acids were the main constituients^16^. Based on this, the fatty acids have been identified in other extracts as responsible for the corrosion mitigation on metal surfaces. Its formula chemical is $\text{R-COOH}$, where R represents the alkyl chain. Its long carbon chain and its feature of having a hydrophilic head (polar) and a hydrophobic tail (nonpolar) are the main features that confer good anticorrosive properties (Fig. 2), e.g., the Hexadecanoic acid known as palmitic acid has a carboxylic group (COOH^−^), which represents the hydrophilic part in the molecule, which can be adsorbed via ${\text{C}}{\text{u}}^{+}$ and ${\text{C}}{\text{u}}^{2+}$ oxide states to form copper carboxyl on the metal surface. Meanwhile, the alkyl chain’s methyl group (${\text{C}}{\text{H}}_{3}$) represents the hydrophobic part that repeals the ${\text{H}}_{2}{\text{O}}$ molecules from the metal surface. The compounds found in the GC/MS chromatogram confirm the observed in the FT-IR where functional groups such as ${\text{C}}{\text{H}}_{3}$ and ${\text{COO}}$H^−^ were detected.

**Table 2:** GC/MS data of the hexane extract of PSN.

Peak	RT	Molecular formula	Compound	Area (%)
1	6.37	C_10_H_16_	Bicyclo[3.1.0]hexane, 4-methylene-1-(1-methylethyl)	6.7
2	18.97	C_18_H_34_O_2_	Ethyl 9-hexadecenoate	6.64
3	19.21	C_18_H_36_O_2_	Hexadecanoic acid, ethyl ester	100
4	19.36	C_16_H_32_O_2_	n-Hexadecanoic acid	62.25
5	20.77	C_20_H_36_O_2_	Linoleic acid ethyl ester	7.78
6	20.85	C_20_H_38_O_2_	Ethyl oleate	64.32
7	22.67	C_18_H_34_O_2_	Oleic acid	25.13
8	22.86	C_13_H_26_O_2_	Undecanoic acid, ethyl ester	27.85
9	40.19	C_15_H_26_O	Humulane-1,6-dien-3-ol	25.39

### Open circuit potential

**Figure 3:**
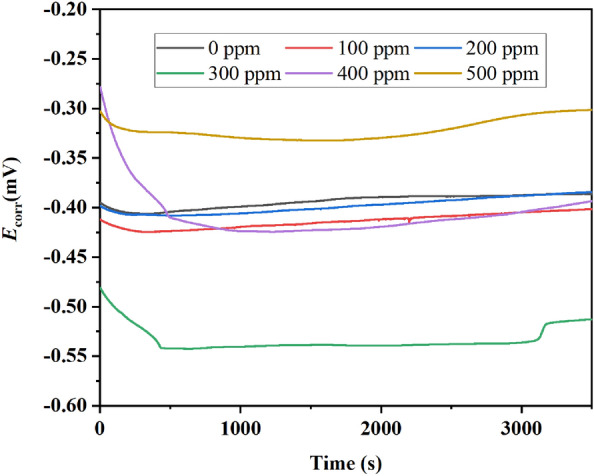
OCP behavior for brass immersed in 0.5 M HCl, without and with hexane extract of PSN as corrosion inhibitor at different concentrations.

The behavior of the corrosion potential (*E*_corr_) for the brass immersed in 0.5 M HCl without and with hexane extract of PSN is shown in Fig. 3. It is clear that for the first 200 s, all measurements present a decrease in OCP potential during the first seconds due to a dissolution of the corrosion film formed on the surface^21^, after which it becomes stable due to a rapid increase in hydrogen ion reduction reactions, after which the anodic and cathodic reactions become equivalent^22^. Then, without PSN (0 ppm), The *E*_corr_ for the brass held a stable behavior of − 0.385 ± 0.059 mV until the end of the test. With the inhibitor addition at 100 ppm, the *E*_corr_ of the brass shifted to more active potentials, meaning an increase in the corrosion process with a stable behavior in its *E*_corr_ of − 0.402 ± 0.063 mV. With the concentration increased at 200 ppm, the *E*_corr_ slightly changed toward a nobler direction with a value of − 0.376 ± 0.073 mV. The most active behavior was registered at 300 ppm, where the PSN helped to shift the *E*_corr_ toward more active values of − 0.533 ± 0.053 mV, suggesting an increase in the corrosion rate. This could be because the compounds contained in the PSN could interact with the active sites on the brass, increasing the corrosion in the areas susceptible to ${\text{C}}{\text{l}}^{-}$ diffusion and avoiding the adsorption of the inhibitor over the brass surface.

At 400 and 500 ppm of PSN, the *E*_corr_ was displaced again in the noble direction (407 ± 0.029 and 321 ± 0.068 mV, respectively). After 3000 s, the OCP values observe an increase, which is due to the thickening of the passive film on the metal surface^23,24^. Then, based on the behavior observed with the inhibitor addition, the PSN suppresses both anodic and cathodic reactions, suggesting it acts as a mixed corrosion inhibitor.

### Electrochemical impedance spectroscopy (EIS)

**Figure 4:**
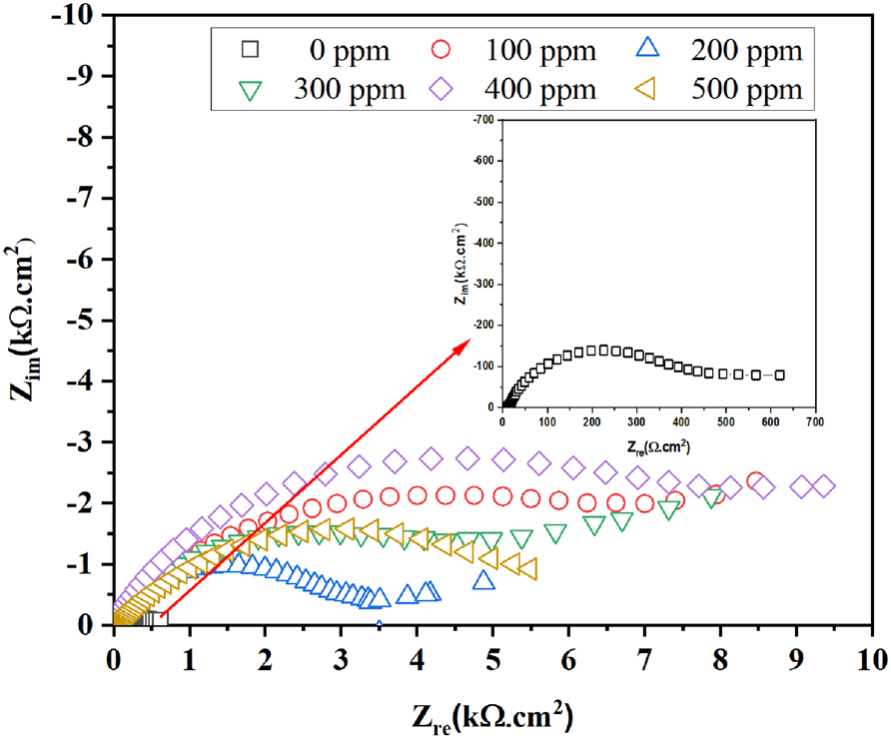
Nyquist plot for the brass immersed in 0.5 HCl, without and with hexane extract of PSN as corrosion inhibitor at different concentrations.

**Figure 5:**
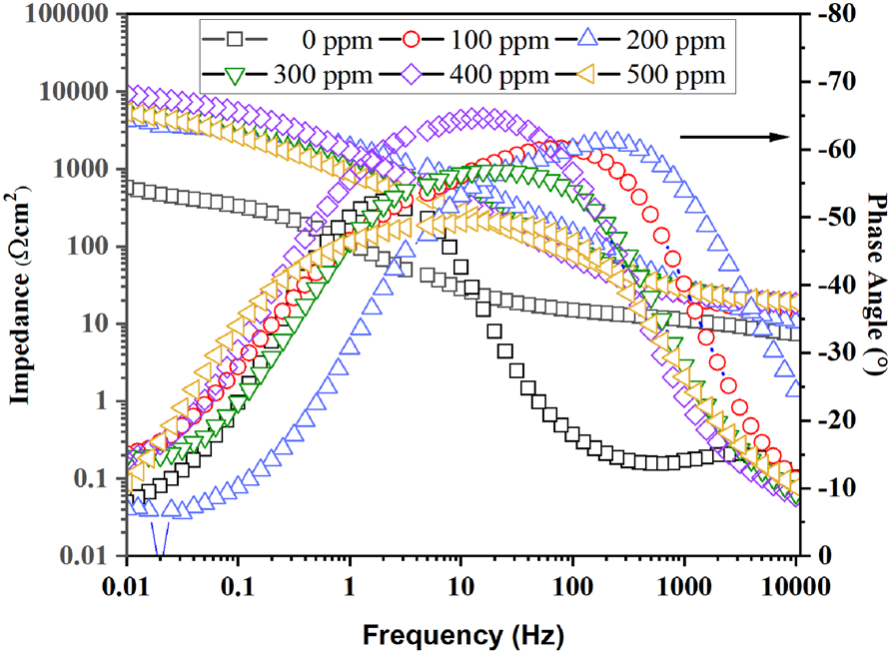
Bode plots for the brass immersed in 0.5 M HCl, without and with hexane extract of PSN as corrosion inhibitor at different concentrations.

Figure 4 shows the EIS plots for the brass without and with PSN immersed in 0.5 M HCl. With the addition of PSN, the semicircle diameter increased from high to middle frequencies with a displacement of the constant phase element toward high frequencies, which can be appreciated in Fig. 5. Also, an elongation for the time constant at this frequency range was appreciated for all PSN concentrations. The resistance increase observed with the presence of PSN is due to the carboxyl groups contained, which have long chain lengths in the hydrophilic part that form complexes with the ${\text{C}}{\text{u}}^{2+}$ ions; behavior reported by Badr et al. when they modified a chitosan surfactant with different hydrophobic chain with which performance in the corrosion resistance on steel was improved^25^. The depression observed in the Nyquist plots can depart from different reasons, such as roughness, impurities, and adsorption of corrosion products, suggesting a non-ideal capacitive behavior. Based on the latter, the test without inhibitor showed a depressive semicircle from high to middle frequencies due to the charge transfer resistance from the electrolyte to the metal surface with a phase angle of − 52° at 2 Hz (middle frequencies). At 100 ppm, an increase in the depressive semicircle was observed with a diffusion attempt at low frequencies, observing a displacement in its phase angle toward high frequencies (> 100 Hz) with a value of − 60° (Fig. 5). This suggested that the PSN was adsorbed on the metal surface, avoiding oxygen and chloride diffusion on active brass sites, leaving it clear that the charge transfer resistance controls the corrosion process. At 200 ppm, a reduction in the semicircle diameter was observed, with a diffusion attempt of Cl^−^ and O atoms into the metal surface. Its phase angle decreased from − 60° to − 53°, displacing to higher frequencies with the inhibitor concentration increases, suggesting a decrease in the covered degree (*θ*) of the PSN on the brass surface. This is because, with the concentration, a change in how the inhibitor is adsorbed on the brass surface occurs, changing from horizontal to vertical, taking place a non-homogenous cover of the surface^26^. At 300 ppm, the semicircle diameter increased from high to middle frequencies, increasing the impedance magnitude and decreasing the phase angle (− 56°) with a displacement of the time constant from high to middle frequencies. A diffusion intent was observed at low frequencies. However, the predominant mechanism continues to be the charge transfer resistance. At 400 ppm, the semicircle diameter increased, reaching its maximum, with an increase in the phase angle of (− 64°). Its impedance magnitude reaches a value of 10^4^ Ωcm^2^, one order more than the test without PSN, which was 10^2^ Ωcm^2^. At 500 ppm, the semicircle diameter decreased again, keeping the total impedance of the brass in the order of 10^3^ Ωcm^2^, suggesting a non-linear behavior of the resistance with the inhibitor concentration.

**Figure 6:**
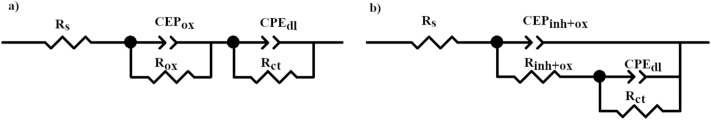
EEC to fit the experimental data from the brass immersed in 0.5 M HCl without (**a**) and with (**b**) hexane extract of PSN as corrosion inhibitor.

To explain the impedance behaviors of the brass without and with PSN as corrosion inhibitors at different concentrations, the experimental data were fitted with the electrical equivalent circuits shown in Fig. 6. The electrical equivalent circuit (EEC) of Fig. 6 a was used to fit the experimental data from the test without inhibitor, where the first element consists of a constant phase element (*CPE*_ox_) at high frequencies, which corresponds to the layer of corrosion products formed over the metal surface configured in parallel with an oxide resistance (*R*_ox_). After, it was connected in series with a constant phase element corresponding to the double layer formed between the metal surface and the electrolyte (*CPE*_dl_). This latter, in turn, was connected in parallel with a charge transfer resistance (*R*_ct_). With the inhibitor addition, Fig. 6b, a *CPE*_ox+inh_ element is added in parallel with a *R*_inh+ox_, and a *CPE*_dl_ was connected in parallel with an R_ct_. Both, in turn, were in series with the *R*_inh+ox_. The *CPE* parameters calculated with the EEC fit are the admittance (*Y*_0_) and the n parameter. To give a better physical explanation of CPE, it can be converted to capacitance values (*C*_x_) with Eq. (1)^27,28^. In the case of n values, these are related to the surface roughness, where some authors suggested that values near 1 mean a more homogeneous surface. Meanwhile, values near 0 suggest a more heterogeneous surface^29^. Also, these values near 1 or near 0 mean an ideal (capacitor) and non-ideal (resistor) capacitive behavior, respectively. Meanwhile, values near 0.5 mean a finite or infinite Warburg diffusion^30^. The EIS efficiency was calculated with Eq. (2), where ${R}_{p}^{0}$ is the polarization resistance without PSN, and ${R}_{p}$ is the polarization resistance at different PSN concentrations.

1 $$C_{x} = (Y_{0} R_{x}^{1 - n} )^{1/n}$$

2 $$\% IE_{EIS} = 100\left[ {1 - \frac{{R_{p}^{0} }}{{R_{p} }}} \right]$$

Table 3 shows the parameters calculated with the EECs. The blank’s total polarization resistance (*R*_p_) was 554 Ωcm^2^, and its *R*_ox_ got a value of 13 Ωcm^2^. Meanwhile, its *R*_ct_ got a value of 536 Ωcm^2^, suggesting that the surface protection was due to the charge transfer resistance. With the presence of PSN in the electrolyte at 100 ppm, the *R*_p_ increased at least one order with 9025 Ωcm^2^, observing a synergistic effect via adsorption of the PSN with the oxide products formed on the brass. At 200 ppm, the *R*_p_ got a value of 4492 Ωcm^2^, observing that the *R*_inh+ox_ increased its value at 3040 Ωcm^2^. At 300 and 400 ppm of PSN in the solution, the *R*_p_ values increased at 8680 and 9878 Ωcm^2^, reaching inhibition efficiencies above 90%. As was discussed before, at 500 ppm, the *R*_p_ decreased at this concentration with an inhibition efficiency of 84%. The n_inh+ox_ values observed tend to increase from 0.5 for the test without PSN to values nearer to 1 for the tests with PSN (between 0.6 and 0.8), suggesting the presence of a metal surface more homogeneous with a non-significant change in its heterogeneity^31^. Also, based on Qiao et al., this behavior suggests the formation of corrosion products more stable on the metal surface due to their interaction with the inhibitor. On the other hand, the n_dl_ observed a decrease in the n values from 0.9 for the test without inhibitor to values near 0.5, indicating the breakdown of the passive layer and some diffusion process formed on the metal surface^32,33^.

**Table 3:** Fitting results from eis analyses for the brass immersed in 0.5 M HCl without and with hexane extract of PSN as corrosion inhibitor.

C_inh_ (ppm)	Chi-Sqr	*R*_s_ (Ωcm^2^)	*C*_inh+ox_ (Fcm^−2^)	n_inh+ox_	*R*_inh+ox_ (Ωcm^2^)	*C*_dl_ (Fcm^−2^)	n_dl_	*R*_ct_ (Ωcm^2^)	*R*_p_ (Ωcm^2^)	%IE
0	1.7 × 10^–3^	5	2.0 × 10^–5^	0.5	13	8.4 × 10^–4^	0.9	536	554	
100	4.6 × 10^–5^	9	5.7 × 10^–7^	0.6	8	1.3 × 10^–5^	0.9	9008	9025	90
200	1.0 × 10^–3^	7	3.4 × 10^–5^	0.7	3040	4.5 × 10^–3^	0.7	1446	4493	80
300	1.4 × 10^–3^	18	9.3 × 10^–5^	0.7	4516	1.9 × 10^–3^	0.6	4146	8680	90
400	3.6 × 10^–4^	19	8.4 × 10^–5^	0.8	5496	3.8 × 10^–4^	0.5	4363	9878	91
500	2.7 × 10^–4^	17	1.2 × 10^–4^	0.7	1491	1.2 × 10^–4^	0.7	4291	5799	84

### Potentiodynamic polarization (PP)

**Figure 7:**
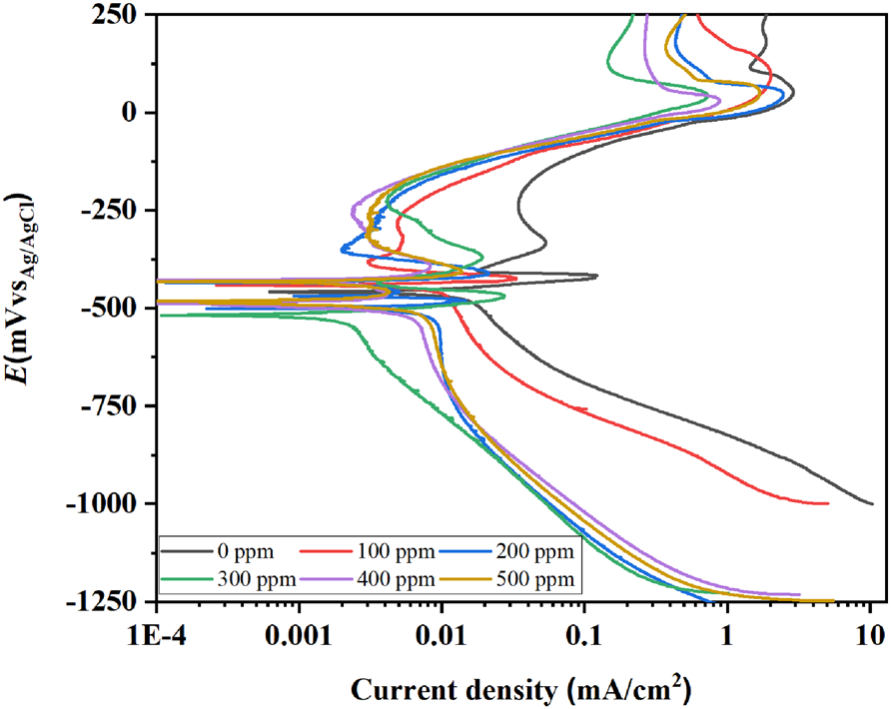
Potentiodynamic polarization curves for the brass at different concentrations of hexane extract of PSN as corrosion inhibitor immersed in 0.5 M HCl.

The potentiodynamic polarization (PP) curves done to the brass immersed in 0.5 M HCl without and with PSN are shown in Fig. 7. The parameters of PP curves were calculated via the Tafel extrapolation method, extending asymptote lines around ± 300 mV dec^−1^ in the Tafel region for both anodic and cathodic branches. The intersection points between these lines allowed the estimation of the *i*_corr_. Finally, to determine the *E*_corr_, a straight line is extended parallel to the current density scale and perpendicular to the potential scale until it intersects the met point of the two lines previously drawn^34,35^. The results are shown in Table 4. All plots present an active–passive behavior, with a decreased corrosion rate with the PSN concentrations. The lower corrosion current density (*i*_corr_) was at 200 ppm of PSN with 2.1 × 10^–3^ mA cm^−2^, followed by the concentration at 400 ppm with 2.4 × 10^–3^ mA cm^−2^. The remaining concentrations got values relatively higher in the same order. The PSN's inhibition efficiency (IE) at all concentrations was above 90% and was calculated with Eq. (3), where ${i}_{corr}^{inh}$ and ${i}_{corr}$ are the corrosion current in the presence and absence of PSN. Also, with the PSN addition at different concentrations, the critical current density passivation (*i*_cp_) was displacement toward lower values against the blank. Their passivation current densities (*i*_p_) observed the same behavior, indicating the formation of a passive layer without significative changes in their passivation potentials (*E*_p_), which means that the mass transfer and energy consumption kept stable, controlling the corrosion reaction on the brass surface with the PSN adition^36^. The corrosion potential (*E*_corr_), without PSN, has a value nobler against the tests with PSN, which increases or decreases based on its concentration toward active or noble values, suggesting that the PSN blocks both anodic and cathodic processes on the brass surface as was confirmed in the OCP analyses which present similar trends with different values against *E*_corr_, this could be due to that the potential measured in OCP is in the function of the immersion time without external current meanwhile polarized condition in PP curves can lead to the hydrogenation of the surface due to the long polarize applied^37,38^. The latter allowed the Cl^−^ ion diffusion into the metal surface formed complex in the active sites promoting the formation of pits via ${\text{CuC}}{\text{l}}^{-}$ and ${\text{Cu}}{\text{Cl}}_{2}^{-}$ dissolution on the surface. An active region followed by a passive zone confirms that a layer of corrosion products is formed on the metal surface. Although the latter is observed even without PSN, the inhibition effect at all concentrations is appreciated in both: *i*_cp_ reduction and primary passive potential (*E*_pp_) increases, this latter tends to shift in the active direction, suggesting that the PSN acts, increasing the active dissolution of ${\text{Cu}}$ to ${\text{C}}{\text{u}}^{2+}$, adsorbing on the corrosion products layer on the brass allowing get *i*_p_ values in the order of 10^–3^ mA cm^−2^. Also, the transpassive potentials (*E*_tr_) were more active with the PSN as corrosion inhibitor.

3 $$IE\% = \left( {\frac{{I_{corr}^{inh} - I_{corr} }}{{I_{corr} }}} \right)*100$$

**Table 4:** Potentiodynamic polarization data for the brass at different concentrations of hexane extract of PSN as corrosion inhibitor immersed in 0.5 M HCl.

*C*_inh_ (ppm)	*i*_corr_ (mA cm^−2^)	*E*_corr_ (mV)	*i*_cp_ (mA cm^−2^)	*E*_pp_ (mV)	*i*_pit_ (mA cm^−2^)	*E*_pit_ (mV)	*i*_p_ (mA cm^−2^)	*E*_p_ (mV)	*E*_tr_ (mV)	EI%
0	3.4 × 10^–2^	− 463	1.1 × 10^–1^	− 416	1.9 × 10^–2^	− 403	5.1 × 10^–2^	− 330	− 192	
100	3.1 × 10^–3^	− 441	3.2 × 10^–2^	− 427	3.1 × 10^–3^	− 379	3.1 × 10^–3^	− 379	− 276	91
200	2.1 × 10^–3^	− 506	1.2 × 10^–2^	− 478	1.3 × 10^–3^	− 469	–	–	− 345	94
300	3.5 × 10^–3^	− 518	2.7 × 10^–2^	− 466	3.7 × 10^–3^	− 431	4.2 × 10^–3^	− 239	− 222	90
400	2.4 × 10^–3^	− 483	4.0 × 10^–3^	− 469	2.4 × 10^–3^	− 429	3.3 × 10^–3^	− 340	− 244	93
500	3.0 × 10^–3^	− 488	4.1 × 10^–3^	− 456	1.4 × 10^–3^	− 429	3.1 × 10^–3^	− 330	− 239	91

### Electrochemical noise (EN)

**Figure 8:**
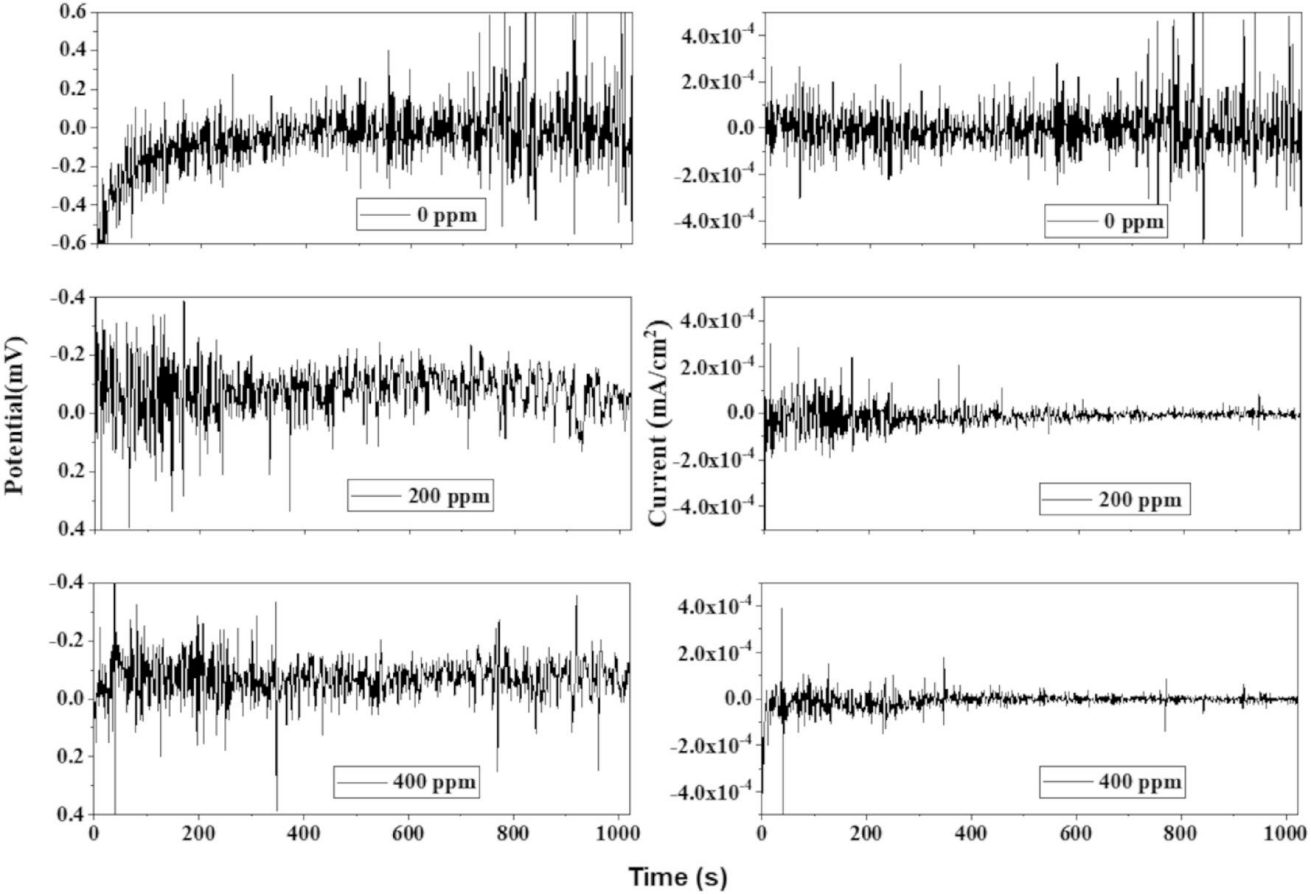
Time series for the potential and current of the brass without and with *Persea schiedeana* Nees (PSN) as corrosion inhibitor immersed in 0.5 M HCl.

Time series from EN for the brass immersed in 0.5 M HCl in the absence and presence of PSN are shown in Fig. 8. The electrochemical potential noise (EPN) signal without PSN (0 ppm) shows an increase in the corrosion potential from active to noble potential values, keeping stable throughout the time with some transients of high amplitude (± 0.6 mV) at 600 s, suggesting an increase in the energy consumption in the mass transfer. In the case of the electrochemical current noise (ECN), the signal remains stable with some transients of low amplitude around ± 2.0 × 10^–4^ mA cm^−2^. After 600 s, their amplitude increased above ± 4 × 10^–4^ mA cm^−2^, suggesting the formation of pits on the brass surface, indicating a localized corrosion type. With the PSN addition (200 ppm), an increase in EPN signal is observed during the first 200 s, which confirms that PSN increases the active dissolution of ${\text{Cu}}$ to ${\text{C}}{\text{u}}^{2+}$ due to the high energy consumption in the mass transfer. However, the EPN transients decreased afterward, keeping stable with transients of low amplitude until the test's end. Its corresponding ECN signal reveals that the current at the beginning of the test showed transients of high amplitude and low frequency decreasing after 200 s from ± 2 × 10^–4^ to ± 1 × 10^–4^ mA cm^−2^, with transients of low amplitude and low frequency, suggesting a generalized corrosion type due to the PSN adsorption on the metal surface helping to produce a more stable layer of corrosion products that avoid the H_2_O interaction with the metal surface due to the hydrophobic part contain in the oleic and palmitic acids compounds found in the PSN extract. The same behavior was observed at 400 ppm of PSN, where a reduction in the ECN signal was appreciated again with a few localized events over time. These results agree with the PP and EIS analyses, where low current densities and high polarization resistances were observed at these PSN concentrations against the test without PSN due to the carbonyl group adsorption on the copper surface, where previous research suggested that this behavior is dependent on the number of carbonyl groups per inhibitor molecule^39^.

Some statistical approaches were conducted to determine parameters such as noise resistance (*R*_n_), skewness, and kurtosis. To obtain these values, it is necessary to first calculate the standard deviation of the current and potential (*σ*_x_) with Eq. (4).

4 $$\sigma_{x} = \sqrt {\frac{{\sum\nolimits_{1}^{N} {\left( {xi - \overline{x} } \right)^{2} } }}{N}}$$

With the standard deviation of the current (*σ*_i_) and potential (*σ*_v_), it is possible to obtain the noise resistance (*R*_n_) by applying Eq. (5).

5 $$R_{n} = \frac{{\sigma_{v} }}{{\sigma_{i} }}$$

To determine the corrosion type on the brass surface, skewness and kurtosis were calculated with Eqs. (6) and (7), where the first refers to the skewed data and the second refers to the outliers found in the EN data measured. Some Skewness and Kurtosis values in the current (*I*_skew_ and *I*_kurt_, respectively) and potential (*E*_skew_ and *E*_kurt_, respectively) have been proposed by Jáquez-Muñoz et al*.* as a reference to determine the type of corrosion present in a metal surface. These values are used here, and the results are shown in Table 5^40^.

6 $$skewness = \frac{1}{N}\sum\limits_{i = 1}^{N} {\frac{{\left( {x_{i} - \overline{x} } \right)^{3} }}{{\sigma^{3} }}}$$

7 $$kurtosis = \frac{1}{N}\sum\limits_{i = 1}^{N} {\frac{{\left( {x_{i} - \overline{x} } \right)^{4} }}{{\sigma^{4} }}}$$

**Figure 9:**
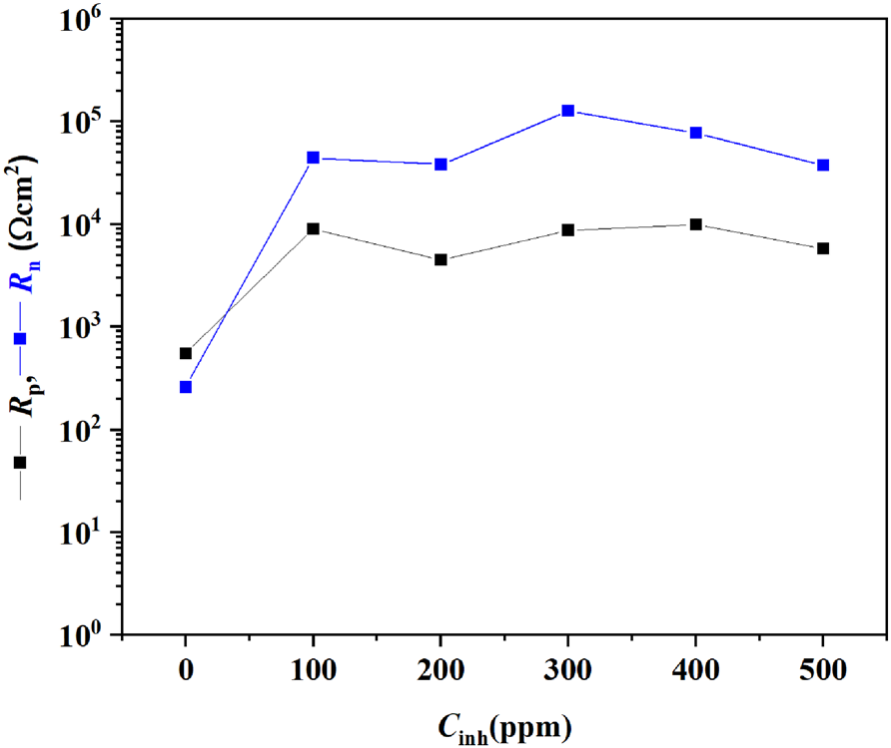
*R*_n_ and *R*_p_ behavior for the brass without and with hexane extract of PSN as corrosion inhibitor immersed in 0.5 M HCl.

The noise resistance (*R*_n_) estimated denotes that the brass resistance tends to increase with the PSN addition into the solution. For the solution without PSN, an *R*_n_ of 10^3^ Ωcm^2^ was calculated. Meanwhile, at 100 ppm, the *R*_n_ increased at 7434 Ωcm^2^. With the inhibitor addition at 200 ppm, a decrease in the *R*_n_ was recorded with 6590 Ωcm^2^. This behavior was like that observed in the EIS test at this concentration. At 300 and 400 ppm, the *R*_n_ increased again at 18,018 and 11,823 Ωcm^2^, respectively. At 500 ppm, a drop in the *R*_n_ was observed, suggesting that the optimal PSN concentration is below this. Figure 9 describes better the *R*_n_ and *R*_p_ behavior, where both have similarities in trend and magnitude.

**Table 5:** Parameters estimated from the ECN and EPN time series for the brass via statistical approaches.

*C*_inh_ (ppm)	*R*_n_ (Ωcm^2^)	σ_i_ (mA cm^−2^)	*E*_kurt_		*E*_skew_		*i*_kurt_		*i*_skew_	
0	103	1.72 × 10^–3^	7	Pitting	2.0		2.0	Uniform	0.8	Uniform
100	7434	8.24 × 10^–4^	2	Uniform	− 0.2	Uniform	1.8	Uniform	− 0.4	Pitting
200	6590	3.70 × 10^–3^	2	Uniform	− 0.1	Uniform	1.8	Uniform	0.5	Uniform
300	18,018	8.26 × 10^–4^	2	Uniform	− 0.3	Uniform	3.1	Pitting	0.6	Uniform
400	11,826	1.75 × 10^–3^	2	Uniform	− 0.1	Uniform	4.7	pitting	1.7	pitting
500	6526	3.27 × 10^–3^	3	Pitting	1.2	Uniform	3.0	Pitting	1.1	Pitting

### Adsorption isotherm for the hexane extract of PSN

The coverage degree (*θ*) should be estimated for the isotherm analysis by substituting the *R*_n_ values in Eq. (8).

8 $$\theta = \frac{{R_{n}^{0} - R_{n}^{i} }}{{R_{n}^{0} }}$$

where $R_{n}^{0}$ and $R_{n}^{i}$ are the noise resistance without and with PSN as corrosion inhibitor, respectively.

**Figure 10:**
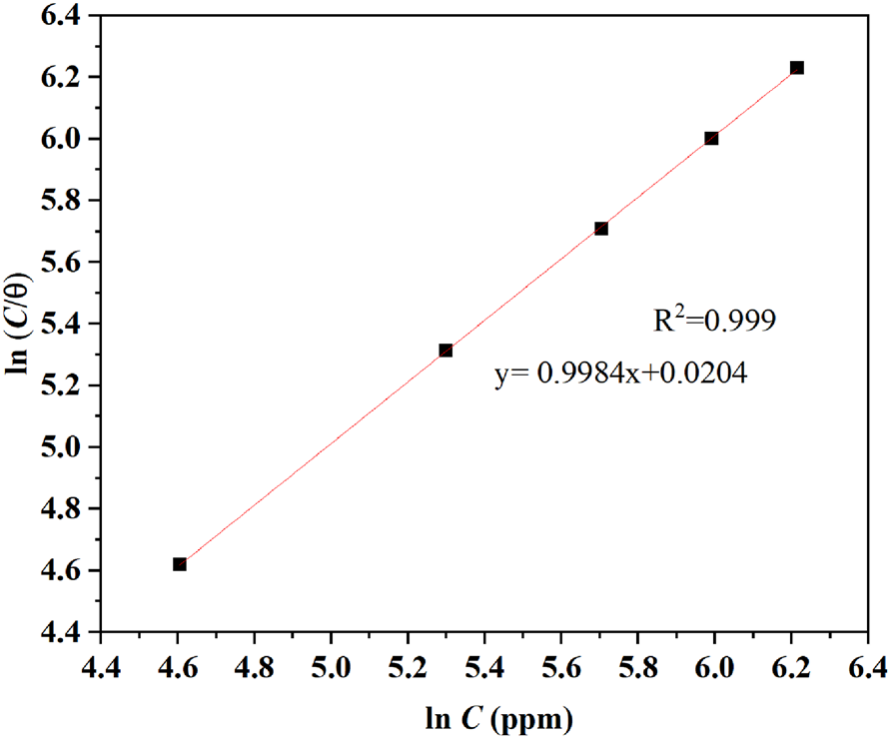
Langmuir adsorption isotherm for the hexane extract of PSN on brass surface immersed in 0.5 M HCl.

Adsorption models were fit using the *θ* values calculated with Eq. (8) versus the inhibitor concentrations used in the bulk solution. To fit the *θ* values, a least square regression line technique was used to obtain the correlation coefficient (R^2^), the slope, and the interception of the data plotted. Some of the models tested were Temkin, Flory Huggins, and Freundlich, which got an R^2^ lower than 0.5. Also, the Langmuir isotherm model in its linear form described in Eq. (9) was used to fit the model, from where an R^2^ of 0.999 was calculated between the model’s prediction (red line) and the experimental data approximation suggested a good correlation between them meaning also an added of the PSN on the brass surface. Also, a slope near 1 (0.9984) was estimated via plotting ln (C/θ) vs. ln C (Fig. 10). This parameter suggested that the inhibitor is adsorbed, obeying this model isotherm, which suggested that each molecule in the PSN occupies one active site in the metal-ion interface without interaction between them. An equilibrium constant (*K*_ads_) of 0.02 was calculated from the reciprocal of the intercept estimated from ln (C/θ) vs. ln C. This value was used to determine the Gibbs Free Energy ($\Delta {\text{G}}_{{{\text{ads}}}}^{0}$) at 25 °C using Eq. (10), got a $\Delta{\text{G}}_{\text{ads}}^{0}$ of − 51.98 kJ mol^−1^ which suggest chemisorption of the PSN compounds since based on the literature values above − 40 kJ mol^−1^ means chemisorption reaction, involving charge sharing or transfer between an inhibitor and metal surface; based on this, the PSN and the Brass forming co-ordinate covalent bonds^41^. Meanwhile, Δ ${\text{G}}_{\text{ads}}^{0}$ negative values suggest that PSN adsorption is spontaneous.

9 $$\ln \left( {\frac{C}{\theta }} \right) = \ln C - \ln k$$

10 $$\Delta G_{ads}^{0} = - {\text{ RT}}ln \, 55.5\left( {\text{k}} \right)s$$

Based on the compounds found by GC/MS, where the main constituents were fatty acids. These can be reacted with the ${\text{C}}{\text{u}}^{2+}$ and ${\text{O}}{\text{H}}^{-}$ ions produced on the anodic (Eq. 11) and cathodic (Eq. 12).

11 $$Cu \to Cu^{2 + } + 2e^{ - }$$

12 $$2e^{ - } + 2H_{2} O \to H_{2} + 2OH^{ - }$$

Then the 2OH- react with the fatty acids to form carboxylate ions (${\text{RCO}}{\text{O}}^{-}$) as is mentioned:

13 $$2OH^{ - } + 2RCOOH \to 2H_{2} O + 2RCOO^{ - }$$

Now, The ${\text{C}}{\text{u}}^{2+}$ formed from the anodic reaction (Eq. 11) can react with the carboxylate ions to form copper carboxylate as follows:

14 $$Cu^{2 + } + 2RCOO^{ - } \to Cu(RCOO^{ - } )_{2}$$

The reaction from Eq. (14) leave clears the interaction between the metal surface and the PSN extract, which is added to the surface-formed complexes in the interface metal-PSN inhibitor via chemical adsorption, decreasing the metal dissolution and avoiding the ${\text{H}}_{2}{\text{O}}$-metal interaction due to the alkali chain in the fatty acid ligand which is hydrophobic part in the fatty acid were other authors such as Tokura et al. found that the carboxylic acid group acts attached on the copper surface reducing the copper oxidation due to its hydrophobic part^42^.

Moreover, the ${\text{C}}{\text{l}}^{-}$ ions in the solution can react with the Cu to promote the formation of unstable compounds such as ${\text{CuC}}{\text{l}}^{-}$ and ${\text{Cu}}{\text{Cl}}_{2}^{-}$ (Eqs. 15, 16), which increase the metal dissolution due to Cl^−^ diffusion. However, it can also occur that ${\text{C}}{\text{u}}^{2+}$ could be produced via ${\text{CuC}}{\text{l}}^{-}$ and ${\text{Cu}}{\text{Cl}}_{2}^{-}$ dissolution (Eqs. 17, 18) and then ${\text{C}}{\text{u}}^{2+}$ react with the ${\text{O}}{\text{H}}^{-}$ ions to form ${\text{CuO}}$ or ${\text{C}}{\text{u}}_{2}{\text{O}}$ (Eqs. 19, 20), highly protective products, improving the corrosion resistance of the brass surface.

15 $$Cu^{ + } + Cl^{ - } \to CuCl^{ - }$$

16 $$CuCl^{ - } + Cl^{ - } \to CuCl_{2}^{ - }$$

17 $$CuCl^{ - } \to Cu + Cl^{ - } + e^{ - }$$

18 $$CuCl_{2}^{ - } \to Cu^{2 + } + Cl^{ - } + e^{ - }$$

19 $$Cu + 2OH^{ - } \to Cu(OH)_{2} \to CuO + H_{2} O$$

20 $$Cu^{2 + } + 2OH^{ - } \to 2Cu(OH)_{2} \to Cu_{2} O + H_{2} O$$

At this estate, the cupric and cuprous oxide can react again with the carboxyl group found in the primary constituents from the hexane extract of PSN to form copper-inhibitor complexes, as described in Eq. (14).

### Corrosion product morphologies

**Figure 11:**
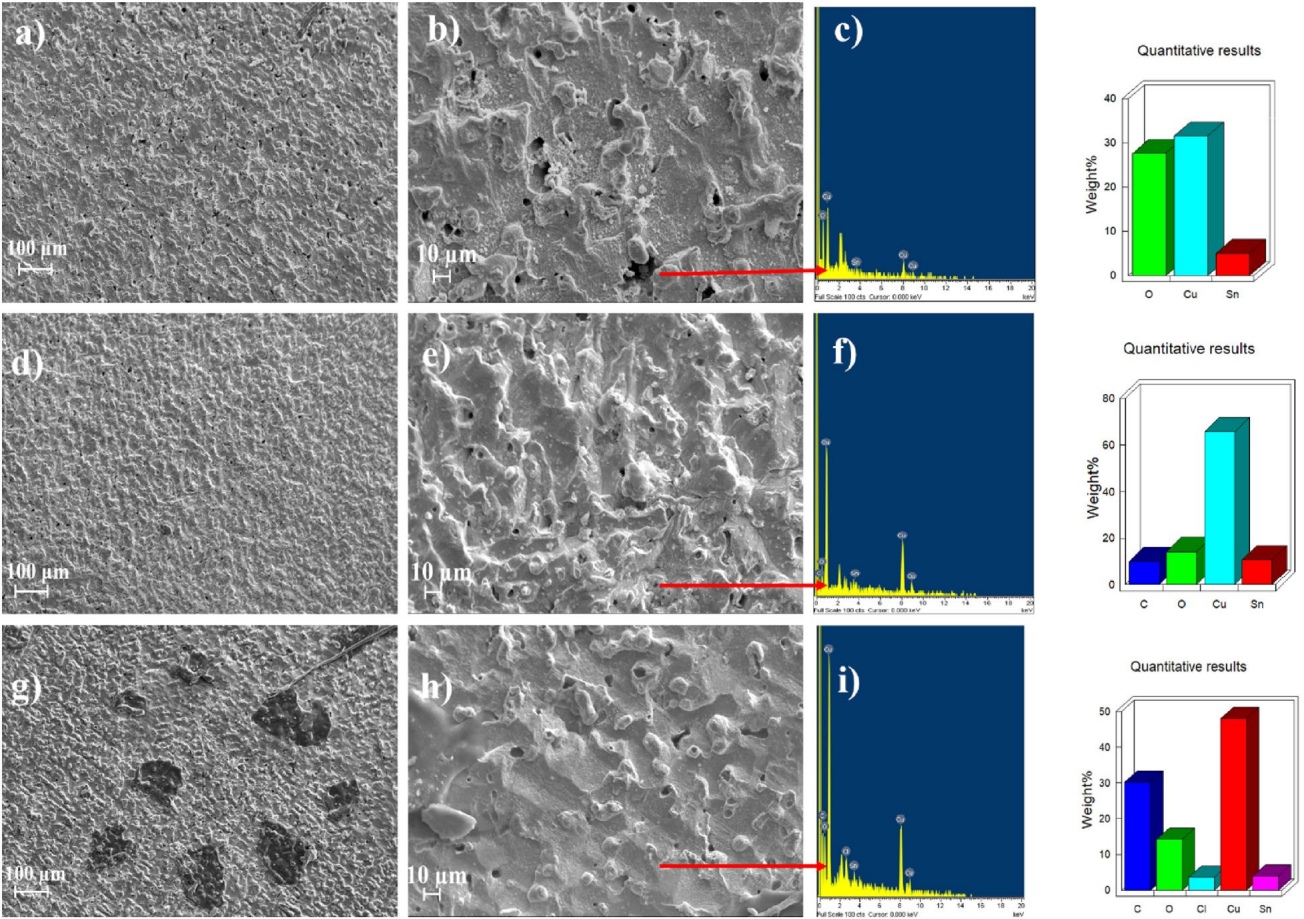
Corroded surface and EDS analyses for the brass without (**a**–**c**) and with hexane extract of PSN at 100 (**d**–**f**) and 200 ppm (**g**–**i**) immersed in 0.5 M HCl.

SEM micrographs and energy dispersive spectroscopy (EDS) are shown in Fig. 11. Figure 11a,b, represent the metal surface without PSN in the solution. The panoramic view (Fig. 11a) shows pits with a diameter of 7 ± 2 µm. Also, a corrosion product scale with an irregular form is present along the metal surface. Figure 11b shows a zoomed view of the corroded surface where the presence of pits on the metal surface generated by the diffusion of ${\text{C}}{\text{l}}^{-}$ ions into the porous forming ${\text{CuC}}{\text{l}}^{-}$ and ${\text{CuC}}{\text{l}}_{2}^{-}$ increased the localized corrosion, generating a detachment on the scale formed by ${\text{CuO}}$ and ${\text{C}}{\text{u}}_{2}{\text{O}}$ from the metal surface. Panoramic views of the brass surfaced exposed at 100 and 200 ppm of PSN concentrations are shown in Fig. 11d,g, respectively, where fewer porosities are observed on the brass surface with an average diameter of 5 ± 2 µm. The zoom views for the brass at 100 and 200 ppm of PSN (Fig. 11e,h, respectively) show the corrosion products layer with fewer zones susceptible to localized corrosion due to the presence of the copper carboxylates in the solution, which helps to avoid the Cl^−^ diffusion into the metal surface due to the barrier formed by the PSN. The EDS analyses for each sample were done to determine the elements on the brass corroded surfaces. They have shown that for all samples, oxygen was detected (Fig. 11c,f,i). Also, the carbon compound is not detected in the test without PSN. Meanwhile, carbon is detected in the tests with PSN at 100 and 200 ppm. The latter suggested the presence of the inhibitor on the metal surface based on the reactions previously described in Eqs. (13–20)^43^.

### Atomic force microscopy (AFM)

**Figure 12:**
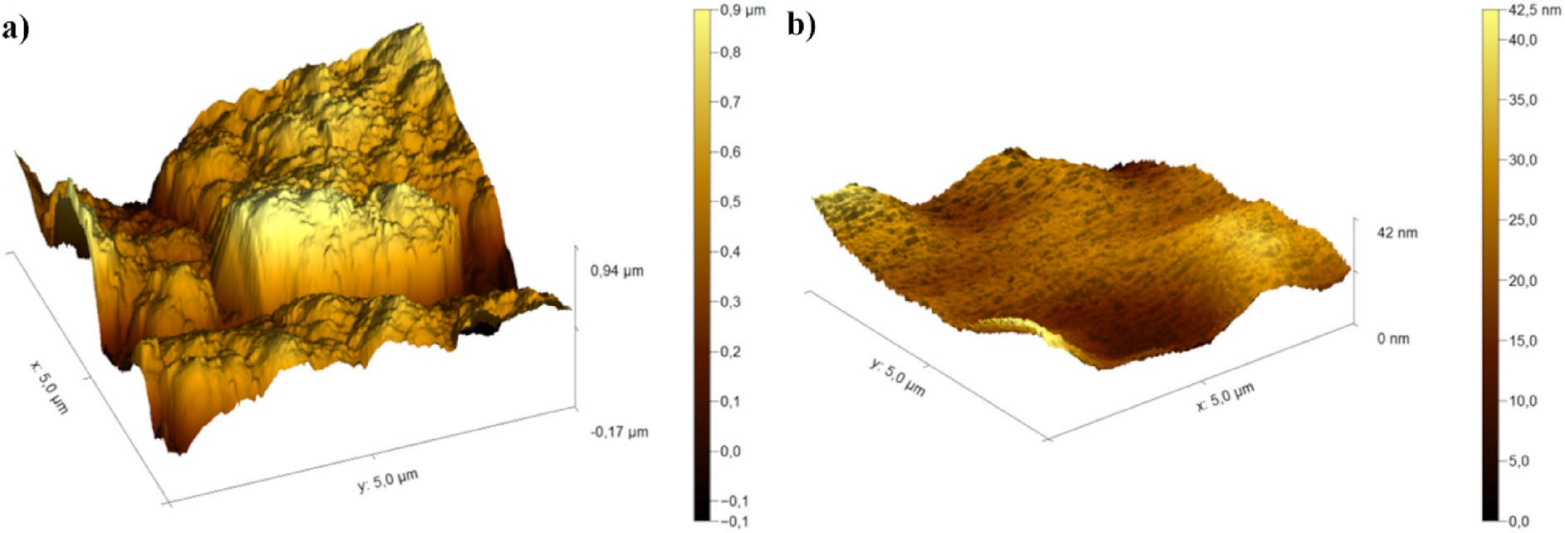
AFM characterization in 3D for the brass surface without (**a**) and with (**b**) PSN.

The AFM characterization in 3D for the brass surface after immersion in 0.5 M HCl without and with PSN is shown in Fig. 12. The 3D analysis for the surface without PSN (Fig. 12a) showed peaks and valleys around 0.94 µm with an average roughness (Sa) of 139.53 nm and a root mean square roughness (Sq) of 180.19 nm. With the PSN addition at 100 ppm, the 3D surface of brass (Fig. 12b) presents less heterogeneity, suggesting the formation of a passive film with peaks and valleys less high of 42.49 nm. Also, the average roughness, indicative of PSN presence on the surface, was 4.5 nm, lower than the Sa in the test without PSN. Moreover, its Sq value was 5.96 nm (Fig. 12).

## Conclusions

Based on the results obtained from the electrochemical test, it was possible to determine that the PSN works as a suitable corrosion inhibitor with efficiencies above 90% in almost all concentrations. The efficiency calculated from EIS and PP analyses confirms that the PSN reduces the corrosion rate on the brass reaching the best performance at 100 and 400 ppm of PSN. The ECN and EPN time series suggested a mixed corrosion type on the metal surface based on skewness and kurtosis values estimated via statistical approaches. Moreover, the amplitude of the transients in the ECN signal decreased with the inhibitor addition from ± 2 × 10^–4^ to ± 1 × 10^–4^ mA cm^−2^ for the test without and with PSN, respectively. Even the *R*_n_ calculated from EN got the same trend as the *R*_p_ estimated from EIS with its maximum noise resistance at 400 ppm of PSN with 11,826 Ωcm^2^. The GC/MS analyses to characterize the PSN hexane extract allowed the identification of the primary compounds in the PSN such as Oleic Acid (25.13%), Humulane-1,6-dien-3-ol (25.39%), Undecanoic acid, ethyl ester (27.85%), n-Hexadecanoic acid (62.25%), and Ethyl Oleate (64.32%). where the two last represent the main constituents in the extract. As was explained, based on the chemical structure of the fatty acid identified, they have a carboxyl group joined to an alkyl chain where the first can form copper carboxylate with the brass surface ($\text{Cu(RCO}{\text{O}}^{-}{\text{)}}_{2}$), allowing the PSN molecule to keep adsorbed over it. Also, the alkyl chain has a methyl group (${\text{C}}{\text{H}}_{3}$) that works as a repellent from the H_2_O molecules, reducing their interaction with the metal surface. Also, the IR analysis confirmed the presence of the functional groups found in the chromatograms. The brass corroded surface showed the presence of carbon with the inhibitor addition which is an indicative of the PSN adsorption on the metal surface. Also, the presence of Cu, O and Cl suggest the formation of corrosion products such as ${\text{CuC}}{\text{l}}^{-}$, ${\text{CuC}}{\text{l}}_{2}^{-}$, ${\text{CuO}}$ and ${\text{C}}{\text{u}}_{2}{\text{O}}$ on the brass surface. AFM characterization showed that the average roughness on the brass surface decreased from 139.53 nm for the blank solution to 4.5 nm in the presence of PSN.

## Data Availability

The authors confirm that the data supporting the findings of this study are available within the article.
